# Cryopreservation of biological materials: applications and economic perspectives

**DOI:** 10.1007/s11626-025-01027-0

**Published:** 2025-04-23

**Authors:** Suja Aarattuthodi, David Kang, Sanjay Kumar Gupta, Paula Chen, Bethany Redel, Moureen Matuha, Haitham Mohammed, Amit Kumar Sinha

**Affiliations:** 1 Plant Genetics Research Unit, United States Department of Agriculture - Agricultural Research Service, Columbia, MO 65211 USA; 2 Biological Control of Insects Research Laboratory, United States Department of Agriculture - Agricultural Research Service, Columbia, MO 65211 USA; 3 Indian Institute of Agricultural Biotechnology, Garhkhatanga, Ranchi, Jharkhand 834003 India; 4 Department of Agriculture and Environmental Sciences, Lincoln University of Missouri, Jefferson City, MO 65101 USA; 5 Department of Rangeland, Wildlife and Fisheries Management, Texas a&M University, College Station, TX 77843 USA; 6 Department of Aquaculture and Fisheries, University of Arkansas Pine Bluff, Pine Bluff, AR 71601 USA

**Keywords:** Cryopreservation, Cryoprotectants, Vitrification, Viability, Functionality, Biobanking, Cryotherapeutics

## Abstract

Cryopreservation is a transformative technology that allows for the long-term storage of biological materials by cooling them to extremely low temperatures at which metabolic and biochemical processes are effectively slowed or halted. Cryopreservation utilizes various techniques to minimize ice crystal formation and cellular damage during freezing and thawing processes. This technology has broad applications in the fields of medicine, agriculture, and conservation, spanning across stem cell research, reproductive and regenerative medicine, organ transplantation, and cell-based therapies, each with significant economic implications. While current techniques and their associated costs present certain challenges, ongoing research advancements related to cryoprotectants, cooling methods, and automation promise to enhance efficiency and accessibility, potentially broadening the technology’s impact across various sectors. This review focuses on the applications of cryopreservation, research advancements, and economic implications, emphasizing the importance of continued research to overcome the current limitations.

## Introduction

Cryopreservation is a cornerstone of modern science enabling the storage and preservation of a wide range of biological materials including cells, tissues, and organs. The fundamental principle behind cryopreservation is to effectively halt all biochemical and metabolic processes to keep the biospecimens in a state of suspended animation (Belzer and Southard 1988; Meryman 2007; Pegg 2007; Taylor 2007; Fahy and Wowk 2015; Jang *et al*. 2017; Whaley *et al*. 2021). The ability to store biomaterials at extremely low temperatures ensures their viability and functionality for future use, which is crucial for advancements in multiple sectors. Cryopreservation is vital for a variety of applications, ranging from medical treatments to biological research and agricultural practices.

Central to the success of cryopreservation are cryoprotective agents (CPAs), which are chemical agents designed to protect biological materials from the damaging effects of freezing (Rall and Fahy 1985; Fuller 2004; Hopkins *et al*. 2012; Sieme *et al*. 2016; Elliott *et al*. 2017). The cryoagents function by preventing ice crystal formation within cells, which can cause mechanical damage and disruption of cellular structures. Common CPAs, such as dimethyl sulfoxide (DMSO) and glycerol, stabilize cellular membranes and maintain cell viability during the freezing and thawing processes (Friedler *et al*. 1988; Mazur *et al*. 2000; Wowk and Fahy 2002; Hunt *et al*. 2003; Fahy *et al*. 2006; Baust *et al*. 2017). The diversity of CPAs available enables tailored approaches for different cell types and applications, continually improving the efficacy and safety of cryopreservation practices.

Cryopreservation stands as a pivotal technology with remarkable potential across diverse sectors. Its capacity to preserve biological materials at ultra-low temperatures is poised to revolutionize health care, agriculture, and conservation, offering transformative benefits and opportunities for growth (Hubálek 2003; Prentice and Anzar 2011; Banker *et al*. 2017; Jang *et al*. 2017; Bojic *et al*. 2021; Nagel *et al*. 2021; Whaley *et al*. 2021; De Falcis *et al*. 2022; Parihar *et al*. 2023; Brister *et al*. 2024; Yan *et al*. 2024). In healthcare, cryopreservation is critical for preserving cells, tissues, and organs for transplantation (Totsuka *et al*. 2002; Taylor 2007; Zhan *et al*. 2022a, b; Ishizaki *et al*. 2023; Sheng and Huang 2024; Han *et al*. 2023; Xue *et al*. 2024; Ozgur *et al*. 2024; Frye *et al*. 2024). Stem cells can be preserved for future use in regenerative medicine and cancer treatment (Heng *et al*. 2005; Fahy *et al*. 2006; Xu *et al*. 2009; Holm *et al*. 2010; Hornberger *et al*. 2019; Chang *et al*. 2021; Parihar *et al*. 2023; Wang *et al*. 2023). The ability to cryopreserve embryos and oocytes has revolutionized reproductive medicine (Walters *et al*. 2009; Banker *et al*. 2017; Iussig *et al*. 2019). Innovations in cryoprotectants and preservation techniques promise to enhance the efficacy and accessibility of these services, potentially reducing costs and improving patient care.

Biotechnology also benefits immensely from cryopreservation, particularly in the field of genetic engineering and in the development of pharmaceuticals (Hubálek 2003; Guo *et al*. 2020; Kuang *et al*. 2022). Microorganisms, cell lines, tissues, etc. used in research and drug production can be stored indefinitely, ensuring the stability and availability of critical biological resources allowing for long-term studies and experimental reproducibility (He *et al*. 2020; Chang *et al*. 2021; Cui *et al*. 2021; Zhang *et al*. 2021a, b; Tutrina and Zhurilov 2024). Additionally, the preservation of genetic material allows scientists to maintain and manipulate rare and valuable lineages and microbial strains with precision (Curry 2000; Purdy 2006).

In agriculture, cryopreservation ensures food security by preserving genetic diversity in crops and livestock. It enables the storage of seeds, sperm, and embryos, which is essential for breeding programs and biodiversity preservation (Curry 2000; Purdy 2006; Seki and Mazur 2009; Engelmann 2011; Wigger *et al*. 2021; De Falcis *et al*. 2022; Yan *et al*. 2024). Future advancements could lead to the development of more resilient and high-yielding varieties, bolstering agricultural productivity and enabling more efficient responses to environmental challenges. In conservation, this technology holds the key to preserving endangered plant and animal species and restoring ecosystems addressing the growing need for effective environmental management (Prentice and Anzar 2011; Pence and Bruns 2022; Mooney *et al*. 2023).

Advancements in cryopreservation procedures have significantly improved its efficiency. Innovations such as vitrification, which involves rapid cooling to prevent ice crystal formation, and controlled-rate freezing techniques that minimize cellular damage have enhanced the preservation of delicate biological samples (Taylor *et al*. 2004; Kuleshova *et al*. 2007; Li *et al*. 2010; Isachenko *et al*. 2012; Banker *et al*. 2017; Wowk *et al*. 2018). Recent developments include the use of nanotechnology and novel CPA formulations to further mitigate ice crystal-related damages and improve outcomes for cryopreserved samples (Etheridge *et al*. 2013). Cryopreservation continues to evolve with advances in CPAs and techniques, enhancing its effectiveness and expanding its range of applications.

Recently, there has been some success with the cryopreservation of complex tissues such as blood vessels, cartilage, and corneas (Fahy *et al*. 2006; Gavish *et al*. 2008; Wang *et al*. 2014; Wowk *et al*. 2018). However, limited success with the cryopreservation of whole organs and other composite tissues using current approaches points to the need for continued research in this area. The development of methods to cryopreserve complex tissues without loss of function would revolutionize several areas of medicine and biomanufacturing. Such a capability would alter the practice of transplant medicine, trauma treatment, fertility treatment, and the manufacturing of engineered tissues for regenerative medical applications.

Cryopreservation offers invaluable tools for preserving biological materials across various fields. Employing sophisticated CPAs and advanced procedural techniques, researchers and clinicians continue to push the boundaries of possibilities, unlocking new potential in medicine, biotechnology, and conservation. This review highlights the applications, research advancements, and economic impacts of cryopreservation. With the growing significance of cryopreservation in various scientific and medical fields, this review also covers future research directions in cryopreservation and the need for ongoing research to address its limitations and to enhance cost-effectiveness and broader implementation.

## History of cryopreservation

**Figure 1.**
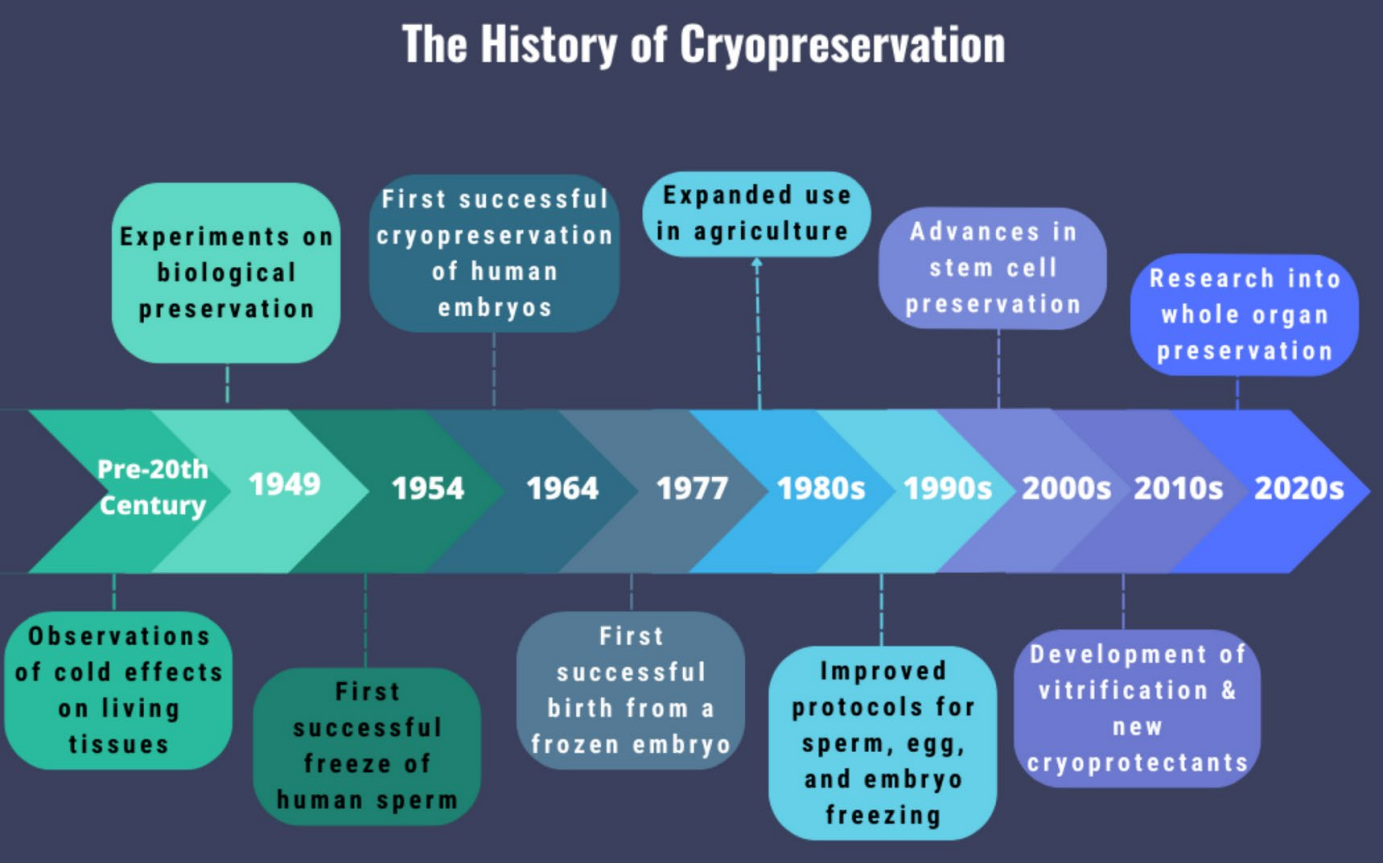
A graphical summary depicting the key events in the history of cryopreservation with the corresponding timelines (Luyet 1937; Polge *et al*. 1949; Gonzales and Luyet 1950; Smith 1950; Bunge and Sherman 1953; Lovelock 1953; Mazur 1963; Farrant 1965; Mazur 1970; Mazur *et al*. 1972; Fahy 1986; Fahy *et al*. 1984; Rall and Fahy 1985; Al-Hasani *et al*. 1987; Chesné and Guillouzo 1988; Friedler *et al*. 1988; Barnes *et al*. 1995; Bouquet *et al*. 1995; Pegg 2005; Fahy *et al*. 2006; Pegg 2007; Walters *et al*. 2009; Fahy 2010; Ock and Rho 2011; Giwa *et al*. 2017; Iussig *et al*. 2019; Whaley *et al*. 2021; Arutyunyan *et al*. 2022; Clavien *et al*. 2022; Zhan *et al*. 2022a, b; Han *et al*. 2023; Ishizaki *et al*. 2023; Wang *et al*. 2023; Filz von Reiterdank *et al*. 2024; Flahaut *et al*. 2024; Ozgur *et al*. 2024).

The history of cryopreservation is a fascinating journey spanning several decades of scientific innovation and discovery (Fig. 1). This field has evolved from rudimentary techniques to sophisticated methods integral to modern biological and medical sciences. From early theoretical ideas to modern techniques that enable the preservation of complex biological systems, cryopreservation has become an indispensable tool in several sectors. The concept of preserving biological tissues via freezing can be traced back to the eighteenth and nineteenth centuries (Pegg 2007). However, these early efforts were rudimentary and largely anecdotal. Though the use of ice to preserve biospecimens was suggested, this idea remained theoretical due to the lack of understanding of the underlying biological processes. In 1776, following the invention of the microscope, Spallanzani observed that sperm could maintain mobility when exposed to cold temperatures (Walters *et al*. 2009). In the late 1800s, researchers used cryopreservation to sustain spermatozoa and red blood cells (Pegg 2007; Whaley *et al*. 2021).

The first significant breakthrough in cell cryopreservation came in the 1940s with the discovery of cryoprotectants. Researchers began to understand that certain substances could prevent ice formation inside cells, which was crucial for preserving cell viability. The protective effects of glycerol in preserving red blood cells laid the groundwork for future research. Breakthroughs by Polge *et al*. (1949) and Smith (1950) demonstrated that cells could survive freezing and thawing when mixed with glycerol, marking the beginning of modern cryopreservation techniques. Glycerol effectively protected the cells from the rapid formation of ice crystals during the preservation process and increased the survivability of spermatozoa (Polge *et al.* 1949; Polge and Rowson 1952). A successful human sperm preservation protocol by freezing and thawing method was developed at the University of Iowa (Bunge and Sherman 1953). This research effort paved the way for the development of the world’s first sperm bank and the first human baby from cryopreserved sperm was born from that pioneering bank. In 1964, the word cryobiology was coined meaning “cold life science.” Cryobiology is the science of how low temperature affects bioactivities and the architecture of biomaterials (Mazur 1970).

The 1950s and 1960s marked a significant advancement in cryopreservation with the development of controlled rate freezing techniques. Another advance occurred in the 1950s with the discovery that the cryopreservation process caused osmotic stress in the cell by instantly freezing the liquid, which led to the formation of damaging ice crystals (Lovelock 1953). In 1963, Mazur characterized the process and reported that the rate of temperature change within a cell controlled the movement of water across the cell membrane and thus the degree of intracellular freezing (Mazur 1963). Researchers further demonstrated that slight increments in the freezing and thawing processes prevented the rapid formation of ice crystals that increased membrane-bound solutes associated with early cell death (Farrant 1965; Mazur *et al*. 1972). Rall and Fahy (1985) made important contributions by developing methods to control the cooling rate of biologics, thus minimizing the formation of ice crystals. During this period, cryopreservation was established as a viable technique for preserving cells and tissues (Pegg 2005; 2007).

The late 1980s and 1990s brought advances in vitrification, a process where cells are rapidly cooled to avoid ice crystal formation, thereby allowing better preservation of cell integrity. This also helped to improve the overall understanding of the mechanism associated with the cryoprotective process. During the 1980s, it was revealed that the rate of freezing and thawing during the cryopreservation process was the most important factor in determining the survivability of the cells (Al-Hasani *et al*. 1987; Chesne and Guillouzo 1988). The pioneering work of Fahy *et al*. (1984) demonstrated that vitrification preserves embryos and other delicate biological materials more effectively than slow-freezing methods.

In the 1990s, researchers developed a range of synthetic cryoprotectants, such as DMSO and ethylene glycol, which significantly improved the efficiency of cryopreservation. These CPAs helped to reduce ice crystal formation and cellular damage during the freezing and thawing processes (Friedler *et al*. 1988; Boquet *et al*. 1995; Ock and Rho 2011). Key contributions in this area came from scientists who explored the mechanisms of CPA action and optimized their use in preserving cells and tissues. In 1995, the first successful transplantation of cryopreserved blastocyst was achieved (Barnes *et al*. 1995). The 2000s onwards saw the integration of cryopreservation techniques with stem cell therapy and regenerative medicine, facilitating advancements in personalized medicine. In the early 2000s, cryopreservation techniques became increasingly important in reproductive medicine (Walters *et al*. 2009; Iussig *et al*. 2019). The ability to successfully preserve and thaw human embryos and oocytes revolutionized in vitro fertilization (IVF) and assisted reproductive technologies (ART). Advances in vitrification techniques, including the development of rapid cooling devices and improved cryoprotectant formulations have further enhanced the success rates of fertility treatments.

Recent years have seen exciting developments in cryopreservation including the use of nanotechnology to improve cryoprotectant delivery and reduce toxicity as well as the exploration of new cryoprotectants and cooling techniques. Researchers are also investigating the potential of cryopreservation for preserving whole organs and complex tissues for transplantation, which could significantly impact the field of organ transplantation.

## Cryoprotectants

Cryoprotective agents are used to protect biological samples from the damage caused by ice crystals formed during the freezing and thawing processes (Fuller 2004; Baust 2006; Acker 2007; Elliott *et al*. 2017). Different CPAs vary in their mechanisms, applications, and effectiveness (Paynter *et al*. 1999; Hunt *et al*. 2003; Inglis *et al*. 2006; Rodrigues *et al*. 2008; Mandumpal *et al*. 2011; Halwani *et al*. 2014; Vian and Higgins 2014). Ever since the cryoprotective effect of glycerol was established, the family of CPAs has been growing and includes cell permeating and non‐permeating agents. Cell permeating agents can enter cells quickly and inhibit the formation of intracellular ice crystals, but have different degrees of toxicity on cells. Non‐permeating agents cannot penetrate the cell membrane but can increase the extracellular osmotic pressure (Wu *et al*. 2005; Rodrigues *et al*. 2008; Chen *et al*. 2011; Murray *et al*. 2024). Proper selection of CPAs is important to improve the viability of the cryopreserved samples. A detailed account of the types of cryoprotectants, mechanisms, doses, types of cells protected, duration of protection, and their advantages and disadvantages are provided (Table 1).

**Table 1.** Different types of cryoprotectants with the mechanisms, concentrations used, types of cells protected, duration of protection, advantages, and disadvantages. This is not an exhaustive list

CPA	Category	Dose	Mechanism	Storage time	Cell types preserved	Advantages	Disadvantages	References
Dimethyl sulfoxide	Small molecule CPA	5–10% (v/v)	Penetrates cell membranes, reduces ice formation, works by disrupting hydrogen bonds in water, stabilizes cellular structures	Long-term (years)	Mammalian cells, stem cells, sperm, oocytes, and embryos	Effective in preventing ice crystal formation; widely used; relatively inexpensive	Toxicity at high concentration or if not removed properly after thawing	Lovelock and Bishop 1959; Friedler *et al*. 1988; Hunt *et al*. 2003; Iwatani *et al*. 2006; Gurtovenko and Anwar 2007; Thirumala *et al*. 2010; Hayakawa *et al*. 2010; Lui *et al*. 2010; Ock and Rho 2011; Mandumpal et al. 2011
Glycerol	Small molecule	5–10%; 10–20% for mammalian cells	Penetrates cell membrane, stabilizes the cellular matrix, reduces ice crystal formation	Short-term and long-term	Bacteria, yeast, some mammalian cells	Low toxicity; effective in protecting a variety of cell types	Osmotic stress and cell damage potential; removal after thawing can be challenging	Mazur 1984; Széll and Shelton 1987; de Backere 1994; Mazur *et al*. 2000; Wowk and Fahy 2002; Fahy *et al.* 2006
Ethylene glycol	Organic compound	1–10% (v/v)	Lowers freezing point and prevents ice formation inside cells	Short-term and long-term	Sperm, embryos	Low toxicity; effective for embryos and a range of applications	Toxic at high concentrations; removal from cells post-thawing can be challenging	Voelkel and Hu 1992; Paynter *et al*. 1999; Dutheil *et al*. 2009; Vian and Higgins 2014
Propylene glycol	Organic compound	1–10%	Similar to ethylene glycol, prevents ice formation by lowering freezing point	Medium-term (months)	Reproductive cells and tissues	Less toxic than DMSO	Limited cell type efficacy	Mazur 1984; Fuller 2004; Vian and Higgins 2014; Murray and Gibson 2022
Trehalose	Disaccharide	0.1–0.5 M (< 0.2 M for mammalian cells)	Stabilizes cellular membranes and proteins during freezing	Long-term (years)	Mammalian cells, embryos, and some plant cells	Non-toxic; good for desiccation; reduces ice formation and enhances cell viability	Less effective compared to other CPAs; relatively expensive; challenging to use with other CPAs	Crowe *et al*. 1984; Aisen *et al*. 2002; Wu *et al*. 2005; Rodrigues *et al*. 2008; Chen *et al*. 2011; Wang and Dong 2017; Murray *et al*. 2024
Hydroxyethyl starch (HES)	Polymer	2–10% (w/v)	Reduces ice formation and stabilizes cell structures during freezing	Medium-term (months)	Embryos, stem cells	Good for tissue preservation	Limited studies; relatively expensive; may cause cellular aggregation	Rowley *et al*. 2003; Elliott *et al*. 2017
Formamide	Organic solvent	1–2 M	Disrupts hydrogen bonds in water, reduces ice formation, and prevents cell dehydration	Medium-term (months)	Some mammalian cells, bacteria	Protects sensitive cells	Toxic to some cell types	Fahy 2010; Best 2015; Elliott *et al*. 2017
Betaine	Organic compound	1–2 M	Protects against osmotic stress and stabilizes cellular membranes during freezing	Medium-term (months)	Some mammalian cells, algae	Non-toxic; protects against osmotic stress	Limited penetration in some cell types	Yang *et al*. 2016; Elliott *et al*. 2017; Murray and Gibson 2022
Sorbitol	Sugar alcohol	1–2 M	Stabilizes cell membranes and prevents dehydration during freezing	Medium-term (months)	Yeast, some mammalian cells	Non-toxic; inexpensive; effective at stabilizing membranes	Can cause osmotic stress at high concentrations	Hubálek 2003; Elliott *et al*. 2017
1,2-Propanediol	Small molecule	1–2 M	Lowers freezing point, protects cellular integrity	Short to moderate	Sperm, tissues	Low toxicity; effective in specific applications	Limited use for sensitive cells	Gook *et al*. 1993; Paynter *et al*. 2001; Katz-Jaffe *et al*. 2008
Sucrose	Sugar	0.5–1 M	Increases osmotic pressure, reduces ice formation	Moderate (weeks)	Embryos, somatic cells	Inexpensive; non-toxic	Limited penetration	Borini *et al*. 2006; Coticchio *et al*. 2006; Rodrigues *et al*. 2008; Sánchez *et al*. 2011
Acetamide	Organic compound	1–10%	Lowers freezing point, alters ice formation	Moderate (weeks)	Oocytes, embryos	Protects during freezing	Limited research available	Fahy 2010; Best 2015; Bartolac *et al*. 2018
2-Methyl-2,4-pentanediol (MPD)	Small molecule	1–2 M	Stabilizes cells, reduces ice crystal formation	Moderate (weeks)	Oocytes, tissues	Low toxicity; effective for oocytes	Not widely used	Pazhang *et al*. 2006; Elliott *et al*. 2017
Dimethylacetamide	Small molecule	1–10%	Stabilizes membranes, reduces ice crystal formation	Short to moderate	Sperm, somatic cells	Good membrane protection	Toxic; limited applications	Fahy 2010; Best 2015; Bartolac *et al*. 2018

### Permeating cryoprotectants

*Dimethyl sulfoxide* is one of the most widely used CPAs due to its ability to penetrate cell membranes and reduce ice crystal formation by lowering the freezing point of water (Friedler *et al*. 1988; Hunt *et al*. 2003; Thirumala *et al*. 2010; Mandumpal *et al*. 2011). It disrupts the hydrogen bonding in water and stabilizes cellular structures during freezing and thawing (Gurtovenko and Anwar 2007). The concentration range of DMSO is 5–10%, which can vary based on the type of cells or tissues being preserved (Boquet *et al*. 1995; Hayakawa *et al*. 2010; Ock and Rho 2011). It is relatively inexpensive and widely used in the preservation of hematopoietic stem cells, animal cells, sperm, oocytes, and embryos (Lovelock and Bishop 1959; Iwatani *et al*. 2006; Lui *et al*. 2010; Ock and Rho 2011). DMSO can provide effective cryoprotection for both short-term and long-term storage, generally ranging from weeks to several years depending on the storage conditions and the specific cells. DMSO is typically mixed with the cell suspension just before freezing. Cells are then cooled at a controlled rate to minimize ice crystal formation and damage. It can be toxic to cells if used at high concentrations or if not properly removed after thawing and requires careful handling to avoid toxicity and to ensure optimal outcomes.

*Glycerol* works by penetrating cell membranes and stabilizing the cellular matrix, thereby reducing the risk of ice crystal formation (Vian and Higgins 2014). This CPA is typically used at 5–10% for bacterial and yeast cells and higher concentrations (10–20%) for mammalian cells. It is less commonly used for complex mammalian cell types compared to DMSO. Glycerol is often used to preserve red blood cells and gametes, and in regenerative medicine (Széll and Shelton 1987; de Backere 1994; Mazur *et al*. 2000; Wowk and Fahy 2002; Fahy *et al*. 2006). It provides effective protection for both short-term and long-term cryopreservation, lasting from several months to years. Cells are usually combined with glycerol and then subjected to controlled-rate freezing or vitrification. It is generally less toxic than DMSO. However, glycerol can cause osmotic stress and damage to cells if not used correctly. Removal of glycerol after thawing can be challenging and requires careful washing.

*Ethylene glycol* prevents ice crystal formation by lowering the freezing point of the solution. It penetrates cell membranes to provide protection during freezing and thawing (Voelkel and Hu 1992; Paynter *et al*. 1999; Dutheil *et al*. 2009; Vian and Higgins 2014). It is used in the preservation of sperm, embryos, stem cells, and other cell lines at a typical concentration of 1–10% (Puts *et al*. 2015). Ethylene glycol is effective for both short-term and long-term cryopreservation with storage times extending from months to years. Ethylene glycol is mixed with cell suspensions before controlled-rate freezing or vitrification. It is less toxic to cells compared to some other CPAs, but potentially toxic at higher concentrations. Removal of this CPA from cells after thawing can be challenging.

*Propylene glycol* reduces ice crystal formation and stabilizes cells during cryopreservation similar to ethylene glycol (Vian and Higgins 2014). It is generally used at a concentration of 1–10% to preserve reproductive cells and tissues (Mazur 1984; Murray and Gibson 2022).

### Non-permeating cryoprotectants

*Polyalcohols* such as trehalose, protect cells by stabilizing cellular membranes and proteins during the freezing process (Crowe *et al*. 1984; Aisen *et al*. 2002; Wu *et al*. 2005; Rodrigues *et al*. 2008; Chen *et al*. 2011; Wang and Dong 2017; Murray *et al*. 2024). It works through a vitrification-like mechanism without forming ice crystals. Typical concentrations range from 0.5 to 1 M and is often used in conjunction with other CPAs to enhance overall effectiveness for cell types sensitive to standard agents. It is used for preserving mammalian cells, embryos, and plant cells (Ahmad and Aksoy 2012). Trehalose provides long-term protection extending from months to years. Trehalose is usually combined with cells in a solution before cryopreservation. It reduces ice formation, enhances cell viability, and is generally less toxic than other cryoprotectants. Higher costs compared to traditional CPAs might render it cost-prohibitive. In addition, it can be challenging to use in combination with other cryoprotectants.

*Hydroxyethyl Starch (HES)* is a polymer that acts as a cryoprotectant by reducing ice crystal formation by means of its viscous properties, which stabilizes the cellular environment during freezing. HES is typically used at concentrations of 2–10% for preserving a variety of cell types including human and animal cells and for stem cell storage (Rowley *et al*. 2003; Elliott *et al*. 2017). HES provides protection for long-term cryopreservation, with effective storage durations of months to years. Cells are combined with HES before freezing using controlled-rate freezing techniques. HES reduces ice crystal formation and cellular damage and is generally well-tolerated by the cells. It may cause cellular aggregation or clumping in some applications and can be expensive compared to other cryoprotectants.

Some of the other CPAs include formamide, acetamide, dimethylacetamide, betaine, sorbitol, sucrose, and 1,2-propanediol (Gook *et al*. 1993; Paynter *et al*. 2001; Borini *et al*. 2006; Coticchio *et al*. 2006; Pazhang *et al*. 2006; Katz-Jaffe *et al*. 2008; Rodrigues *et al*. 2008; Sánchez *et al*. 2011). Additional details on these CPAs are provided in Table 1.

*Extenders:* Cryopreservation of livestock semen, including extender composition, has been extensively investigated to ensure efficient dissemination of important genetics. Traditional extenders have used egg yolk or soybean lecithin, but improvements in post-thaw viability have been seen with the addition of antioxidants (Chanapiwat *et al*. 2009; Akhter *et al*. 2011; Najafi *et al*. 2014; Amidi *et al*. 2016). In bulls, adding vitamin E during cryopreservation increased the number of acrosome-intact spermatozoa and improved motility characteristics (Hu *et al*. 2011). Moreover, the addition of cysteine and glutamine to extender for buffalo semen improved progressive motility and membrane integrity (Topraggaleh *et al*. 2014). Similar results were observed for boar spermatozoa when the extender was supplemented with cysteine, allowing for increased glutathione synthesis (Zhu *et al*. 2022). However, the effects of semen extenders are highly species-specific which warrants further studies in other species.

Specialty CPAs including disaccharides, ice blockers or modulators, and/or natural antifreeze compounds in combination with other CPAs are usually less toxic (Carpenter and Hansen 1992; Koshimoto and Mazur 2002; Matsumura and Hyon 2009; Nishijima *et al*. 2014; Cheung *et al*. 2017; Robles *et al*. 2019). With regard to tissue cryopreservation, permeability and viscosity can stand in the way of obtaining uniform distributions of the CPA solutions to all parts. Natural antifreeze proteins (AFPs) found in cold-adapted fish or frog species are less toxic but prohibitively expensive (Amir *et al*. 2004; Inglis *et al*. 2006; Halwani *et al*. 2014; Kim *et al*. 2017). However, successful cryopreservation using insect-derived AFPs in combination with DMSO has been reported (Halwani *et al*. 2014; Qadeer *et al*. 2016). It has been shown that oocytes and embryos vitrified with AFPs lower ice crystal formation and ensure better survival rates and viability in several animal species (Thiesen and Jordan 2008; Cheung *et al*. 2017; Manuchehrabadi *et al*. 2017).

Adding accessory compounds that mitigate the effects of oxidative damage, osmotic stress, and cryoprotectant toxicity, or augment natural healing processes can increase the efficacy of CPAs. Effective and less toxic cryoprotectant cocktails can be developed by applying computational chemistry, modeling, simulation, and optimization. Additionally, metabolomics, proteomics, genomics, and epigenetics could provide more information to intervene in toxic reactions to cryoprotectants, osmotic stress, and chilling injury. Advanced imaging techniques would help analyze CPA and ice distribution in three-dimensional systems.

Cryoprotectants play a crucial role in the preservation of biologics, each offering unique advantages and limitations. DMSO and glycerol are among the most used CPAs due to their effectiveness and low cost, though they can be toxic at higher concentrations. Ethylene glycol provides a similar function but is less commonly used due to its own set of challenges. Hydroxyethyl starch and trehalose offer alternative methods with specific benefits, though they may be expensive or require specialized handling. Choosing the right cryoprotectant depends on the type of cells or tissues being preserved, the desired duration of storage, and the specific requirements of the cryopreservation process. Cryopreservation continues to evolve with advances in cryoprotectants and techniques, enhancing its effectiveness and expanding its applications. As research progresses, new cryoprotectants and methods are being developed to improve cell survival rates and reduce the risks associated with the preservation process.

## Cryopreservation procedure and techniques

**Figure 2.**
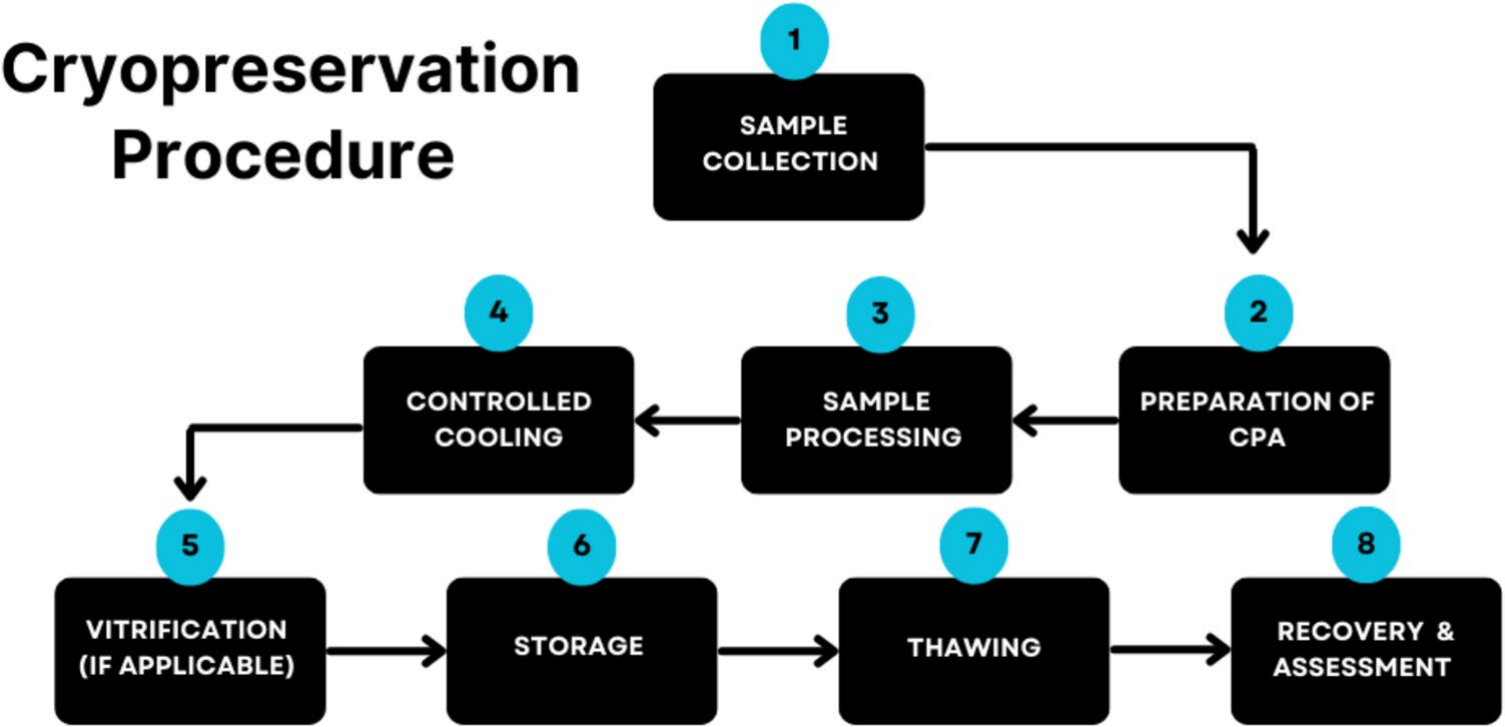
A schematic representation of the cryopreservation process, illustrating the general steps from sample preparation to storage and revival

Different steps in the cryopreservation procedure involve preparation, controlled freezing, thawing, and post-thaw recovery (Fig. 2). In the preparatory stage, cells are suspended in a cryoprotectant solution. The choice of the CPA and its concentration depends on the type of cells and the intended use. Since the cooling rate is critical, during freezing, controlled rate freezers gradually cool the cell suspension to avoid ice crystal formation. A typical protocol involves cooling at a rate of 1 °C/min until reaching − 80 °C, where samples are then stored in the vapor phase of liquid nitrogen at − 196 °C, maintaining the cells in a frozen state. During the revival stage, the cryostocks are thawed rapidly (using a 37 °C water bath) to minimize the formation of ice crystals. After thawing, cells are washed to remove excess cryoprotectant, which can be toxic if left in high concentrations. Cells are then placed in appropriate growth media and conditions to recover from the freezing and thawing process. Post-thaw cell viability is assessed using the Trypan Blue exclusion method or flow cytometry (Strober 2015; Emamverdi *et al*. 2015).

Several cryopreservation techniques are available that ensure the long-term storage of biologics, each with its advantages and disadvantages (Table 2). The primary goal is to preserve viability and functionality during and after the freezing and thawing processes. The selection of technique often depends on the type of biospecimen and the specific requirements for preservation. Some of the methods are mentioned below.

**Table 2:** An overview of various cryopreservation techniques including mechanisms, applications, advantages, and disadvantages

Cryopreservation technique	Mechanism	Type of biomaterials preserved	Equipment required	Suitability for applications	Advantages	Disadvantages	References
Slow freezing	Gradual cooling, minimizes ice crystal formation	Cells, tissues, embryos	Programmable freezer	Reproductive biology, stem cell research	Reduces cellular damage, well-established	Time-consuming, risk of ice crystal damage	Isachenko *et al*. 2004; Borini *et al*. 2006; Boldt *et al*. 2006; Katkov *et al*. 2006; Loutradi *et al*. 2008; Lee *et al*. 2010; Saragusty & Arav 2011; Edgar and Gook 2012; Rienzi *et al*. 2017; Gurruchaga *et al*. 2018; Sevenler *et al*. 2021; Ishizaki *et al*. 2023
Vitrification	Rapid cooling forms a glass-like state	Oocytes, embryos, stem cells	Liquid nitrogen, vitrification solutions	Assisted reproductive technology, cell therapy	Minimal ice crystal formation, high survival rates	Requires precise techniques, expensive reagents	Fahy *et al*. 1984; Wowk *et al*. 2000; Taylor *et al*. 2004; Pegg 2005; Chen *et al*. 2006; Moon *et al*. 2008; Ciotti *et al*. 2009; Sánchez *et al*. 2011; Lane *et al*. 2011; Cheng *et al*. 2014; Best 2015; Fahy and Wowk 2015; Jang *et al*. 2017; Arutyunyan *et al*. 2022; Zhan *et al*. 2022a, b; Han *et al*. 2023; Rosero *et al*. 2024
Freeze-drying (lyophilization)	Removal of water under low temperature and pressure	Proteins, enzymes, biological samples	Freeze dryer, vacuum system	Long-term storage of biomolecules	Extended shelf life, reduces storage volume	Potential protein denaturation, complex process	Pegg 2007; Mokhova *et al*. 2023
Direct liquid nitrogen freezing	Immediate immersion in liquid nitrogen	Sperm, oocytes, cells	Liquid nitrogen tank	Fertility clinics, biobanks	Quick and effective cooling	Risk of thermal shock, uncontrolled cooling	Gonzales and Luyet 1950; Smith 1982; Chen *et al*. 2006
Controlled rate freezing	Computer-controlled cooling system	Tissues, organs, embryos	Programmable freezer	Organ transplantation, regenerative medicine	Precise temperature control, minimizes damage	Costly, requires sophisticated equipment	Ware *et al*. 2005; Ozgur *et al*. 2024
High-pressure freezing	Freezing under high pressure to inhibit ice formation	Cells, tissues	High-pressure freezer	Cell biology, tissue engineering	Reduces ice crystal damage	Specialized equipment, limited availability	Studer *et al*. 2001; Kaech and Ziegler 2014
Ultrasonic freezing	Uses ultrasound to enhance ice nucleation	Cells, tissues	Ultrasonic device	Biomedical research, tissue engineering	Improved freezing uniformity	Limited application, requires specialized equipment	Ma *et al*. 2021; Mokhova *et al*. 2023
Electro-freezing	Utilizes electric fields to enhance cooling	Cells, tissues	Electroporation device	Biotechnology, regenerative medicine	Promotes cell viability	Complex process, requires precise control	Cassone and Martelli 2024
Cryopreservation in nanotubes	Encapsulation in carbon nanotubes to protect against ice damage	Cells, biomolecules	Nanotubes, controlled temperature units	Nanotechnology applications |	Protects cells from ice crystal damage	Complex fabrication process	Ren *et al*. 2020; Huang *et al*. 2021
Supercooling/ chilling	Cooling below the freezing point without crystallization	Liquids, cells, food products	Supercooling chambers	Food preservation, biological samples and research	Reduces crystallization, minimal damage to materials	Limited to certain materials and specific conditions	Berendsen *et al*. 2014; Puts *et al*. 2015; Bruinsma *et al*. 2015; de Vries *et al*. 2019; 2020; Berkane *et al*. 2024; Filz von Reiterdank *et al*. 2024
Microfluidic cryopreservation	Utilizes microfluidics for precise cooling	Cells, tissues	Microfluidic devices	Biomedical applications, research	High control over freezing conditions	Requires specialized fabrication	Pyne *et al*. 2014; Zhao and Fu 2017
Gel-based cryopreservation	Use of gel matrices to stabilize cells during freezing	Cells, tissues	Gel matrices, cryopreservation containers	Tissue engineering, regenerative medicine	Protects cells from damage	Gel formulation can be complex	Inaba *et al*. 1996; Gryshkov *et al*. 2015; Zhang *et al*. 2018a, b; Kang *et al*. 2021; Sevenler *et al*. 2021; Li *et al*. 2024
Hypothermic cryopreservation	Storage at 0–4 °C temperatures to reduce metabolic and degradation rates	Cells, tissues, organs	Cryopreservation containers	Cell biology, regenerative medicine	Reduces ice formation	Limited to certain cell types	Fuller & Lee 2007; Dutheil *et al*. 2009; Day *et al*. 2017
Natural cryopreservation	Utilization of natural protective agents in freezing	Biological samples	Natural cryoprotectant solutions	Food preservation, biological research	Safe and effective	Limited research on efficacy	Carpenter and Hansen 1992; Koshimoto and Mazur 2002; Amir *et al*. 2004; Inglis *et al*. 2006; Matsumura and Hyon 2009; Nishijima *et al*. 2014; Halwani *et al*. 2014; Kim *et al*. 2017; Cheung *et al*. 2017; Robles *et al.* 2019

*Slow freezing* involves gradually lowering the temperature of the biomaterial at a controlled rate, typically 1 °C per minute. This technique uses CPAs to protect cells from ice crystal formation and allows cells to equilibrate with the CPAs. The process is relatively straightforward and requires less sophisticated equipment compared to some other methods. Slow freezing has a well-established protocol for many cell types and tissues, making it a standard practice in various applications (Isachenko *et al*. 2004; Borini *et al*. 2006; Boldt *et al*. 2006; Katkov *et al*. 2006; Loutradi *et al*. 2008; Lee *et al*. 2010; Saragusty and Arav 2011; Edgar and Gook 2012; Rienzi *et al*. 2017; Gurruchaga *et al*. 2018; Sevenler *et al*. 2021; Ishizaki *et al*. 2023). However, the disadvantages are CPA toxicity and potential cell damage, leading to reduced cryoprotection efficiency. Since prolonged exposure to CPAs can be toxic to cells, careful management of concentration and exposure time are critical. In addition, slow freezing creates cryo‐induced injury due to the formation of extracellular ice. Despite controlled cooling, intracellular ice crystal formation or dehydration is possible leading to lower post-thaw viability. The process is relatively slow, which can be another disadvantage when large quantities of samples need to be processed.

*Vitrification* involves ultra-rapid cooling to transition the intracellular fluid into a glass-like state without forming ice crystals (Fahy *et al*. 1984; Song *et al*. 2000; Taylor *et al*. 2004; Lane *et al*. 2011; Jang *et al*. 2017; Zhan *et al*. 2022a, b; Han *et al*. 2023; Rosero *et al*. 2024). Vitrification avoids damage associated with ice crystals and often results in higher post-thaw survival rates for embryos and oocytes. Vitrification requires rapid cooling of the samples into deep cryogenic temperatures with high CPA concentrations (Arutyunyan *et al*. 2022). Despite the advantage of avoiding freeze injury, there are potential downsides, including CPA‐induced cytotoxicity, high risk of pathogenic agent contamination, and high demand for manipulation skills (Best 2015; Fahy and Wowk 2015). However, the rapid cooling and warming process reduces the exposure time to CPAs, minimizing toxicity. Requirements such as precise control over cooling and warming rates and specialized equipment make vitrification more complex than slow freezing. Additionally, not all cell types and tissues are suitable for vitrification and specific protocols must be developed for different biological materials.

Cryopreservation through vitrification was described in the 1980s (Rall and Fahy 1985) and has been employed successfully for the preservation of several types of biologics (Fahy *et al*. 1984; Arav and Zeron 1997; Wowk *et al*. 2000; Taylor *et al*. 2004; Pegg 2005; Chen *et al*. 2006; Moon *et al*. 2008; Ciotti *et al*. 2009; Sánchez *et al*. 2011; Edgar and Gook 2012; Cheng *et al*. 2014). Zhan *et al*. (2022a, b) used vitrification and rewarming for pancreatic islets with high cell survival even after 9 mo of cryogenic storage. A 92% cure rate in diabetes was observed in mice that received these cryopreserved islet cells 24–48 h post-transplantation. The study reported vitrification to be an efficient method for improving the islet supply and transplant outcomes for diabetes treatment. Vitrification of nucleated cells using a cooling rate of 20 K/min has been demonstrated by using a mixture of CPAs to minimize toxicity and the cells showed long-term survival (Rall and Fahy 1985). The application of vitrification in tissues has been difficult to realize due to limitations in diffusive heat, mass transfer, and phase-change causing the procedure to fail. Studies have shown promising results in tissues such as thin veins, blood vessels (Song *et al*. 2000; Taylor *et al*. 2004), small mammalian organs (Gavish *et al*. 2008; Xu *et al*. 2009), and limbs (Wang *et al*. 2014). To preserve bulk tissues more efficiently, vitrification methods need to be further refined (Etheridge *et al*. 2013), which could potentially make vital organs, and ovary banking part of medical practices. It would also enable the storage of 3D-engineered tissues for regenerative medicine (Giwa *et al*. 2017; Petrenko *et al*. 2017).

*Intermediate temperature cryopreservation* involves cooling samples to intermediate temperatures (typically between − 80 and − 150 °C) using isothermal conditions or controlled rate freezers (Wolkers *et al*. 2007; Taylor *et al*. 2019). Liquid nitrogen temperatures are not reached. Intermediate temperatures can reduce ice crystal formation compared to slow freezing, although it is not eliminated. This method may reduce the need for expensive liquid nitrogen storage and therefore lowers cost. However, this technique is less commonly used and is not as well-established as slow-freezing or vitrification. The effectiveness of CPAs can vary and some cells may not survive the intermediate temperatures as well as they do at lower temperatures.

*Freeze drying* (lyophilization) works by the removal of water under low temperatures and pressure and provides long-term storage of biomolecules (Pegg 2007; Mokhova *et al*. 2023). It is a desiccation technique effective in preserving heat-sensitive materials such as proteins, vaccines, and some drugs. In the post-COVID era, the freeze-drying of live human virus vaccines has emerged as an area of critical importance, offering significant advantages in the storage, transportation, and administration of vaccines. The global pandemic underscored the need for rapid and widespread vaccination efforts, and the challenges faced in vaccine distribution highlighted the essential role that freeze-drying can play in overcoming logistical barriers. This dry technique of cryopreservation aids in the storage and transportation of biomaterials without the need for extensive equipment.

Hyper- or hypothermic techniques involve cooling or warming samples to temperatures above or below the typical cryopreservation range, often using specialized equipment to achieve rapid temperature changes (Fuller and Lee 2007; Dutheil *et al*. 2009; Day *et al*. 2017). This allows for the development of tailored protocols that can be optimized for specific types of cells or tissues. Advantages include the preservation of complex tissues or organs. However, it requires sophisticated equipment and precise control, making it more complex and potentially more expensive. These techniques are often less standardized and may require extensive customization for different applications.

Microencapsulation of cells before cryopreserving is a promising approach for future transplantation purposes (Aoki *et al*. 2005; Kang *et al*. 2021). Inaba *et al*. (1996) reported the merits of alginate‐based microencapsulation in pancreatic islets cryopreservation. Further studies have demonstrated that alginate-encapsulated cryopreserved islets provide significant restoration of euglycemia in diabetic mice compared to non-encapsulated counterparts yielding improved success in long-term grafts in rats.

Some of the other cryopreservation methods include controlled rate freezing, direct liquid nitrogen freezing, high-pressure freezing (Studer *et al*. 2001; Kaech and Ziegler 2014), ultrasonic freezing (Alcalá *et al*. 2023), electro-freezing (Cassone and Martelli 2024), gel-based cryopreservation, cryopreservation in nanotubes, supercooling (Berendsen *et al*. 2014; Bruinsma *et al*. 2015; Puts *et al*. 2015; de Vries *et al*. 2019; de Vries *et al*. 2020; Sevenler *et al*. 2021; Li *et al*. 2024; Berkane *et al*. 2024; Filz von Reiterdank *et al*. 2024), hypothermic cryopreservation (Fuller and Lee 2007; Dutheil *et al*. 2009; Day *et al*. 2017), microfluidic cryopreservation (Pyne *et al*. 2014; Zhao and Fu 2017), and natural cryopreservation (Carpenter and Hansen 1992; Koshimoto and Mazur 2002; Amir *et al*. 2004; Inglis *et al*. 2006; Matsumura and Hyon 2009; Halwani *et al*. 2014; Nishijima *et al*. 2014; Cheung *et al*. 2017; Kim *et al*. 2017; Robles *et al*. 2019) (Table 2).

Each cryopreservation technique has its strengths and limitations, making the choice of method dependent on the specific requirements of the biological material being preserved. Slow freezing is well-established and simple but can cause ice crystal damage and toxicity issues. Vitrification offers high survival rates but is complex and involves significant CPA toxicity. Intermediate temperature cryopreservation and hyper- or hypothermic techniques present niche solutions with their own sets of advantages and challenges. Continued advancements and refinements in these techniques are crucial for improving the efficacy, efficiency, and cost-effectiveness of cryopreservation in various applications.

## Cryopreservation of various biological materials

Cryopreservation is highly dependent on the type of biological material and different materials require distinct preservation methods due to their unique properties. Different cell types and tissues respond differently to cryopreservation. In addition, the efficacy of cryopreservation varies among plant and animal species requiring specialized conditions (Towill 2002; Engelmann 2011; Ren *et al*. 2020; Nagel *et al*. 2021). This variability can complicate the development of universal cryopreservation methods. Factors such as cell structure, sensitivity to freezing, and the presence of CPAs must be carefully considered to ensure optimal survival and functionality post-thaw. Developing, validating, and optimizing specific cryopreservation protocols can improve success rates. Research to understand the specific responses of cell types and tissues to cryopreservation can facilitate the development of more effective protocols. As cryopreservation technologies continue to advance, the techniques employed for different materials will likely evolve, offering higher success rates and greater preservation of biological integrity.

*Animal cells* are the most common cryopreserved biological material (Mazur 1984; Lee *et al*. 2010; Flahaut *et al*. 2024; Li *et al*. 2024). Ice crystal formation inside the cells can rupture the cell membrane and damage the intracellular structures. Preservation of cellular integrity is a key goal. Slow freezing is used for cell lines and vitrification for sensitive cell types. Dimethyl sulfoxide, glycerol, ethylene glycol, etc. are the common CPAs. Different types of cells (e.g., lymphocytes, fibroblasts) may have unique sensitivity to freezing and may require specific protocols depending on the cell type. For instance, primary fibroblasts can usually withstand routine cryopreservation techniques without significant decreases in viability (Mohamed *et al*. 2024). However, the viability of endothelial and epithelial cells is typically negatively impacted by cryopreservation methods (Marschalek *et al*. 2023). These cells are relatively more resilient to cryopreservation compared to more complex tissues and organs.

*Plant cells* differ from animal cells due to the presence of a rigid cell wall. This presents an additional barrier to the effective penetration of CPAs, which adds complexity to the cryopreservation process. CPAs used include DMSO, glycerol, and sorbitol, but special formulations are sometimes required. Vitrification is widely used for sensitive plant tissues. Other techniques like slow freezing or encapsulation-dehydration are used for specific plant types (Pence and Bruns 2022). Encapsulation-dehydration method ensures successful cryopreservation of plant cells, where plant tissues are encased in a matrix and moisture is removed before freezing. Plant cells need to be treated with osmotic agents (such as sorbitol) to remove water from the cells, as excess water can form ice crystals and damage the cells (Menges and Murray 2004).

*Gametes (sperm/oocyte)*: While sperm cryopreservation is relatively straightforward, oocyte cryopreservation is more complex due to the oocyte’s large size and sensitivity to freezing (Fabbri *et al*. 2001; Lane *et al*. 2011; Wigger *et al*. 2021; Yan *et al*. 2024). Moreover, certain species, such as pigs, have high lipid content in the oocyte cytoplasm that can hinder freezing ability. Different CPAs (DMSO, glycerol, ethylene glycol, trehalose) and concentrations are required for sperm and oocytes, based on their structure and function. Oocytes are sensitive to high concentrations of CPAs. While slow freezing is the most commonly used method for sperm with storage in vials or straws, vitrification is ideal for oocytes due to their sensitivity. The sperm membrane is delicate and must be protected to prevent rupture during the freezing process. Removal of seminal plasma is often necessary since it can be toxic to the sperm during freezing.

*Blood cells (RBCs, WBCs, platelets)* are cryopreserved for clinical applications such as transfusions. Glycerol, DMSO, and HES are the common CPAs used to preserve cell integrity and to prevent hemolysis. RBCs and platelets have more specific protocols than WBCs due to their sensitivity to freezing (Schindler *et al*. 2004). Slow freezing is commonly used for RBCs, which need to be slowly frozen in CPAs to prevent damage. Platelets may require more specialized methods such as controlled-rate freezing as these are highly sensitive to freezing and thawing. WBCs, especially lymphocytes, are more resilient than RBCs but still require careful cryoprotectant handling.

*Embryonic and adult stem cells* must be preserved in a way that maintains their pluripotency/differentiation capacity and viability (Faulkner and Wilkes 2006; McGrath and Uitto 2008; Wang *et al*. 2023; Rosero *et al*. 2024). Embryonic stem cells (ESCs) are more sensitive to freezing than adult stem cells and therefore require more refined cryopreservation techniques. Vitrification is often used for embryonic stem cells. Slow freezing and controlled-rate freezing are common for adult stem cells such as mesenchymal stem cells and hematopoietic stem cells. Common CPAs used are DMSO, trehalose, glycerol, etc.

*Tissues (skin, muscle, *etc*.)* require specialized cryopreservation methods to preserve cellular and extracellular matrix integrity (Pegg *et al*. 1997; McGann and Karl 2007; Morris and Baust 2008; Taylor *et al.* 2019; Ishizaki *et al*. 2023; Sheng and Huang 2024; Xue *et al*. 2024). Like cells, tissues need perfusion with CPAs to prevent ice crystal formation and damage. Slow freezing or vitrification is used depending on the tissue type (Ishizaki *et al*. 2023). Slow freezing and cryoprotectant perfusion are typically used for tissues like skin or muscle. Larger tissues are more challenging to freeze due to their complex structure.

*Ocular tissues (retina and cornea)* require preservation of cellular integrity and function. The corneal endothelial cells are crucial for maintaining corneal transparency, which are very sensitive to oxidative stress and freezing damage (Armitage 2009). Retina cryopreservation typically requires a more controlled freezing process. DMSO, glycerol, and trehalose are used in the process. Both vitrification and slow freezing are used, depending on the type of the ocular tissue.

*Organs (e.g., kidney, heart, liver)* require relatively advanced cryopreservation protocols including perfusion of CPAs through blood vessels to avoid damage from ice crystals in the vascular system (Fahy *et al*. 2004; McGann and Karl 2007; Clavien *et al*. 2022; Tessier *et al*. 2022; Han *et al*. 2023; Xue *et al*. 2024; Ozgur *et al*. 2024; Frye *et al*. 2024). Larger organs present significant challenges to freeze due to their size and complex structure. The main focus is maintaining organ function and viability post-thaw. Slow freezing with CPA perfusion is the most commonly used method.

## Applications of cryopreservation

**Figure 3.**
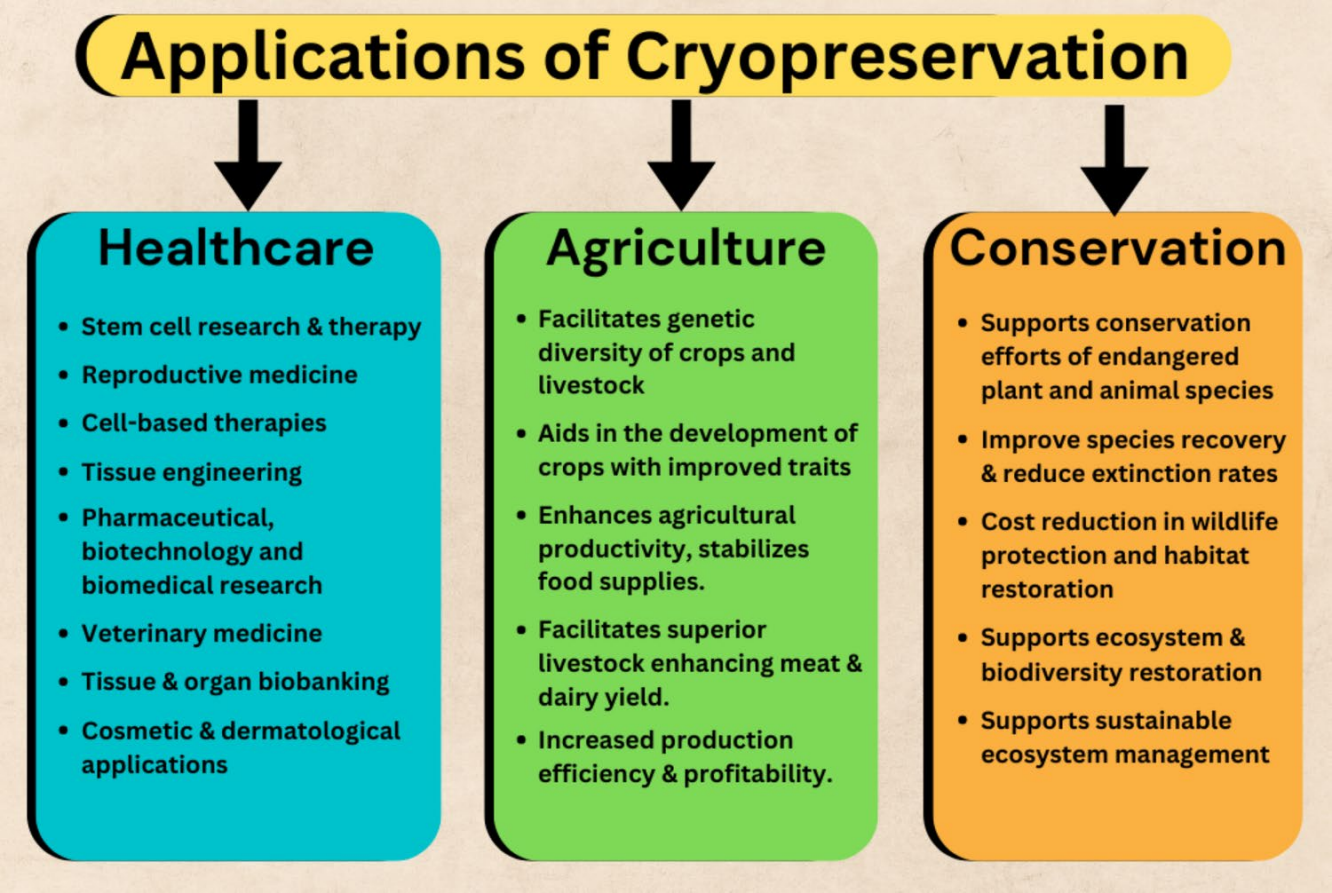
Applications of cryopreservation in the fields of medicine, agriculture, and conservation (Johnston and Lacy 1995; Bernard and Fuller 1996; Holt and Pickard 1999; Curry 2000; Hanks 2001; Totsuka *et al*. 2002; Lockwood 2003; Boettcher *et al*. 2005; Fahy *et al*. 2006; Purdy 2006; Andrabi and Maxwell 2007; Meryman 2007; Simpkins *et al*. 2007; Engelmann 2011; Prentice and Anzar 2011; Bruinsma *et al*. 2015; Lewis *et al*. 2016; Giwa *et al*. 2017; Jang *et al*. 2017; Ward *et al*. 2018; Chang *et al*. 2021; Cui *et al*. 2021; Nagel *et al*. 2021; Zhang *et al*. 2021a, b; De Falcis *et al*. 2022; Kuang *et al*. 2022; Wang *et al*. 2023; Brister *et al*. 2024; Tutrina and Zhurilov 2024; Xue *et al*. 2024; Yan *et al*. 2024; ). This is not an exhaustive list

Cryopreservation has revolutionized many fields including medicine, agriculture, and conservation (Acker 2007; Meryman 2007; Jang *et al*. 2017; Kuang *et al*. 2022; Brister *et al*. 2024) (Fig. 3). It plays a crucial role in stem cell research, regenerative medicine, reproductive technologies, etc. enhancing patient options and outcomes while driving substantial revenue. In agriculture, it is crucial to preserve genetic material from crops and livestock, enabling the development of improved strains and supporting biodiversity. Conservation also benefits from cryopreservation, as it helps maintain the genetic resources of endangered species. Each application presents unique challenges and opportunities. While advancements continue to improve the efficacy of cryopreservation, ongoing research is essential to address the limitations and enhance the applications of this vital technology.

1. *Stem cell research and therapy*: Cryopreservation is crucial for the storage of hematopoietic stem cells used in bone marrow transplants for treating hematological disorders such as leukemia and lymphoma (Buchanan *et al*. 2004; Hornberger *et al*. 2019). Embryonic stem cells are cryopreserved for research and potential therapeutic applications, such as regenerative medicine to treat a variety of conditions, including cancer, spinal cord injuries, and cardiovascular diseases (Heng *et al*. 2005; Fahy *et al*. 2006; Chang *et al*. 2021; Wang *et al*. 2023; Rosero *et al*. 2024). Cryopreservation of induced pluripotent stem cells supports their use in disease modeling and therapeutic research (Holm *et al*. 2010). Stem cells have significant potential and cryopreservation is crucial for their storage and utilization (Xu *et al*. 2009; Parihar *et al*. 2023). The use of cryopreserved stem cells for transplantation not only improves patient survival rates but also minimizes the costs associated with long-term care and hospitalization. Stem cell banks and therapy companies create economic opportunities in the biotechnology sector.
2. *Reproductive medicine*: By preserving high-quality gametes, cryopreservation can enhance the success rates of ART allowing for fertility preservation and IVF potentially reducing the need for multiple cycles and associated costs (Bernard and Fuller 1996; Lockwood 2003; Mukaida 2009; Banker *et al*. 2017). Cryopreservation of gametes enables individuals to delay reproduction before undergoing treatments like chemotherapy (Cobo *et al*. 2016; Iussig *et al*. 2019). Cryopreservation of oocytes contributes to research on reproductive biology, aging, and fertility. As societal trends shift towards delayed childbearing and advancements in cancer treatments increase survival rates, there is a growing demand for fertility preservation services.
3. *Cell-based therapies*: Cryopreservation is integral to the development and application of cell-based therapies for various diseases. In cancer immunotherapy, cryopreservation of T cells and other immune cells is vital (Ci *et al*. 2020). In gene therapy, cells modified with therapeutic genes are cryopreserved to treat genetic disorders. Cryopreservation supports cell-based therapies for autoimmune diseases by storing modified or unmodified cells.
4. *Tissue engineering*: Recent years have witnessed significant progress in tissue regeneration. Biomaterials with native or modified cells are widely used to build tissue‐engineered constructs including skin, bone, cartilage, muscle, and other tissues. The storage of cell‐biomaterial composited products is essential for tissue engineering (Pegg *et al*. 1997; Acker 2007; Urbani *et al*. 2017; Arutyunyan *et al*. 2022). Cryopreserved skin cells are used for grafts in burn victims and patients with skin disorders. Cells used in the regeneration of cartilage and bone tissues are cryopreserved for orthopedic applications. Tissue‐engineered skins are useful in wound healing (De Backere 1994; Bravo *et al*. 2000; Zheng *et al*. 2015). The ability to preserve complex engineered tissues (skin, bone, cartilage, peripheral nerve grafts, blood vessels, connective fascia, adipose tissue, etc.) for extended duration would potentially transform the industry.
5. *Pharmaceutical, biotechnology and biomedical research*: Cryopreservation of microbial cultures and cell lines is crucial for producing biopharmaceuticals (Hubálek 2003; Guo *et al*. 2020; Kuang *et al*. 2022; Tutrina and Zhurilov 2024). Similarly, preservation of plant and animal biomaterials is vital in biotechnology. Preservation of cell lines and tissues allows for long-term studies and experimental reproducibility (Buchanan *et al*. 2004; He *et al*. 2020). Biological organism‐based therapy is a promising direction of biomedicine. Cryopreservation facilitates both inactivated and live organism‐based therapies. For instance, gastrointestinal diseases are treated with cryopreserved fecal microbiota (Cui *et al*. 2021). Also, predatory bacteria are used to eliminate drug‐resistant bacteria. To achieve efficient infiltration of the predatory bacteria, cryo-microneedles (cryoMNs, Chang *et al*. 2021) are used where the bacterial viability remains >80% after the long‐term cryo‐storage. The predatory bacteria encapsulated cryoMNs displayed excellent inhibition effect on Gram‐negative bacteria (Chang *et al*. 2021). In another study using a rodent eye infection model, the infection was significantly reduced in the cryoMN treatment group without altercations in the cornea thickness and morphology (Cui *et al*. 2021). Fungal infections can be treated by predatory bacteria using cryoMNs and ice MNs (Zhang *et al*. 2021a, b).
6. *Veterinary medicine*: Cryopreservation is used to store semen and embryos from valuable livestock, facilitating breeding programs and genetic research. In animal breeding, it helps maintain genetic diversity and improve breeding efficiency. Also, genetic material is cryopreserved to support the conservation of endangered species. Preservation of biological samples over extended periods facilitates long-term studies and reproducibility of experiments.
7. *Tissue and organ biobanking*: Cryopreservation can mitigate organ and tissue shortages by preserving those for longer periods, potentially reducing wait times for transplants and improving patient outcomes (Fahy *et al*. 2006; Simpkins *et al*. 2007). Extended preservation times could lower the costs associated with organ transportation. The feasibility of many transplant and trauma procedures is limited by the time required to transport donor tissues to the recipient (Totsuka *et al*. 2002; Fahy *et al*. 2006; Simpkins *et al*. 2007; Lewis *et al*. 2016; Giwa *et al*. 2017). Many harvested donor organs are discarded because of logistical demands that exceed their storage lifetime. Organ banking supported by cryopreservation addresses organ shortage and the logistical constraints of organ transport. While entire organs are often cryopreserved for short-term, tissues such as skin and corneas are preserved for transplantation (Winship *et al*. 2010; Ishizaki *et al*. 2023). Extending the viability of tissues beyond several hours post-harvest would transform transplantation and reconstructive medicine (Totsuka *et al*. 2002; Simpkins *et al*. 2007; Giwa *et al*. 2017; Han *et al*. 2023; Ishizaki *et al*. 2023; Sheng and Huang 2024; Frye *et al*. 2024; Ozgur *et al*. 2024; Xue *et al*. 2024). The ability to bank organs and tissues would have an immediate impact on transplant medicine, surgical cancer treatment, treatment of combat trauma, and industrial accidents.
8. *Cosmetic and dermatological applications*: Cryopreservation is used in cosmetic procedures and treatments. Cryopreserved skin cells are used in cosmetic and dermatological treatments, including skin rejuvenation and reconstructive surgeries (De Backere 1994; Bravo *et al*. 2000).
9. *Agriculture*: Cryopreservation supports the preservation of genetic material from crops and livestock, facilitating the maintenance of genetic diversity and aiding in the development of resilient crop varieties and livestock breeds that can withstand environmental changes and diseases (Curry 2000; Engelmann 2011; Nagel *et al*. 2021). This in turn enhances agricultural productivity, stabilizes food supplies, and reduces the economic risks associated with crop failures or livestock diseases (De Falcis *et al*. 2022). Cryopreservation of seeds and plant germplasm allows for the long-term storage of crop varieties and aids in the development of new crop strains with improved traits which can lead to substantial economic benefits (Towill 2002). In livestock, sperm cryopreservation facilitates the use of superior genetics across large populations, enhancing meat and dairy production efficiency (Purdy 2006). This practice helps farmers achieve higher yields and better-quality products, contributing to increased profitability.
10. *Conservation*: Cryopreservation of genetic material from endangered plant and animal species supports breeding programs and conservation efforts (Johnston and Lacy 1995; Holt and Pickard 1999; Hanks 2001; Boettcher *et al*. 2005; Andrabi and Maxwell 2007). The costs associated with wildlife protection and habitat restoration are reduced by improving the chances of species recovery (Prentice and Anzar 2011). Enabling the storage of genetic material supports ecosystems and biodiversity. This can reduce the economic impact of environmental degradation and support sustainable ecosystem management. Storage of seeds, sperm, and whole organisms can be crucial for research into the effects of climate change. This also helps in stabilizing food supplies and reducing the economic risks associated with crop failures or livestock diseases. It also fosters opportunities, attracting funding and investments for research and conservation programs contributing to economic growth within the environmental and research sectors.

## Economic implications of cryopreservation

Cryopreservation offers substantial economic benefits across multiple sectors including healthcare, agriculture, and conservation. Additionally, emerging technologies and industries leveraging cryopreservation offer new economic opportunities. The continued advancement of cryopreservation is expected to drive economic growth and efficiency across various fields.

### Healthcare

In healthcare, cryopreservation is revolutionizing organ transplantation, stem cell therapy, and fertility preservation. Cryopreservation supports high-value services including IVF and stem cell therapies contributing to significant revenue streams. The scale of organ transplantation has significantly increased worldwide (Thongprayoon *et al*. 2020; 2022; Zhang *et al*. 2021a, b; Lentine *et al*. 2022; Kwong *et al*. 2023; Frye *et al*. 2024). There were 41,354 organ transplants (a 5.9% increase) performed in the USA in 2021, compared to 2020 (Thongprayoon *et al*. 2022). However, solid organ transplantation still presents challenges (Thongprayoon *et al*. 2020). Cryopreservation of organs and tissues can address critical shortages and potentially reduce the overall costs of transplantation (Papaleo *et al*. 2017). The number of people waiting for organ transplants often exceeds the available supply, leading to higher costs associated with urgent care and prolonged hospital stays (Page and Woodward 2008). Cryopreservation could reduce the logistical and operational costs associated with organ procurement and transportation. Improved preservation techniques are reported to lead to cost efficiencies by minimizing the need for emergency organ retrieval and maximizing the utilization of available organs (Guibert *et al*. 2011).

Cryopreservation of complex tissues and organs has a potential market with direct applicability across the full spectrum of medical treatment and diagnostics (Ward *et al*. 2018; Frye *et al*. 2024). Many harvested donor organs are discarded because of logistical challenges (Nguyen *et al*. 2023). Total costs for heart transplant procedures can be exponentially high with extreme logistical demands that require transplantation within a few hours of organ harvest. Cryopreservation can be a potential solution to these challenges.

Stem cell therapy supported by cryopreserved cells has the potential to revolutionize regenerative medicine driving economic growth. The use of stem cells for treating cancer, spinal cord injuries, cardiovascular diseases, neurological disorders, etc. can lead to significant savings by improving patient outcomes, thereby reducing the costs associated with long-term care and hospitalization. The global market for stem cell therapies is growing rapidly driven by the increasing demand for regenerative medicine. Commercial opportunities exist for stem cell banks and therapy companies creating economic opportunities and jobs in the biotechnology sector.

The demand for cryopreservation in fertility clinics and related services has led to increased revenue and growth in the reproductive medicine sector. Fertility clinics can charge substantial fees for cryopreservation and storage, generating significant revenue. In the USA, the cost of egg freezing can range from $6000 to $15,000 per cycle, with annual storage fees adding to the income of fertility clinics (Roque *et al*. 2017). Fertility preservation services are increasingly in demand due to societal shifts and medical advancements and benefit economically from cryopreservation by enabling individuals to delay or safeguard their reproductive options. This trend supports the economic viability of fertility clinics, which are seeing increased revenues from cryopreservation services (Lockwood 2003).

Cryopreservation supports biobanking availing researchers a steady supply of biologics for drug discovery and disease research (Wigger *et al*. 2021). This not only advances scientific knowledge but also stimulates the biotechnology sector economically. For instance, biobanks can charge for sample access and data creating a revenue stream while facilitating advancements in personalized medicine and pharmaceutical development.

### Agriculture

Cryopreservation supports the preservation of crop and livestock varieties, contributing to food security and economic efficiency (De Falcis *et al*. 2022). By preserving genetic material, cryopreservation supports the development of resilient crop varieties and livestock breeds that can withstand environmental changes and diseases and has a key role in the disaster recovery of crops (Engelmann 2011). This stabilizes food supplies and reduces the economic risks associated with crop failures or livestock diseases. The economic benefits include increased crop and livestock yields. By enabling the preservation and use of genetic material without the need for maintaining large breeding herds, cryopreservation reduces costs associated with animal husbandry and breeding programs.

Cryopreservation aids in the maintenance of genetic diversity, improved crop yields, and enhanced breeding programs leading to increased productivity and profitability for farmers and agribusinesses. The use of cryopreserved germplasm in agriculture has been crucial in developing disease-resistant varieties of wheat and maize, which can lead to substantial economic benefits for the agricultural sector (De Falcis *et al*. 2022). This technology allows for the storage of valuable genetic material for future breeding programs, potentially reducing costs associated with field-based preservation. This also helps in stabilizing food supplies and reducing the economic risks associated with crop failures or livestock diseases.

### Conservation

By preserving genetic material from endangered species, cryopreservation aids breeding programs and species recovery efforts, reducing the economic burden of wildlife protection and habitat restoration (Martino & Kenter 2023). This can also mitigate the economic impact of biodiversity loss, which affects industries like ecotourism and agriculture. Cryopreservation supports conservation programs which in turn benefit local economies dependent on tourism and natural resource management. Cryopreservation can reduce the costs associated with *in-situ* conservation efforts, such as habitat protection and anti-poaching measures. It also attracts funding and investments that bolster the environmental and research sectors.

### Emerging industries

Cryopreservation supports the development and commercialization of bioengineered tissues and organs with the potential to transform healthcare and create new revenue streams. For example, the commercialization of bioengineered skin for burn treatment and cryopreserved tissues for organ transplants represents a growing market with significant economic potential (Bravo *et al*. 2000; Ward *et al*. 2018). Research into nanotechnology for cryopreservation is paving the way for economic opportunities. Nanoparticles used to enhance cryoprotectant efficacy can lead to more effective and cost-efficient cryopreservation techniques (Murray and Gibson 2022). This can result in reduced costs for research and clinical applications, potentially opening new markets and creating economic growth in the biotech sector.

## Future research directions

Cryopreservation with its capacity to preserve biological materials at ultra-low temperatures offers significant promise across diverse fields. However, to fully harness its potential and overcome the limitations, future research must address several key areas.

1. *Innovation and optimization of cryoprotectants*: As some of the traditional CPAs can be toxic at high concentrations, the development of non-toxic/less toxic CPAs is relevant. Research could also focus on improving existing CPAs or their combinations that can reduce cellular damage during the freezing and thawing processes. Investigations into natural CPAs from extremophiles are also promising.
2. *Advances in vitrification*: Advanced vitrification formulations, better techniques to enhance the transition to glass state, novel cooling methods, and controlled warming are under exploration.
3. *Nanotechnology applications*: Nanoparticles can be designed to improve the uniformity of cooling and heating processes and to deliver CPAs more effectively. Nanoparticles might also help in reducing ice crystal formation and minimizing cell damage (Thiesen and Jordan 2008; Etheridge *et al*. 2013; Manuchehrabadi *et al*. 2017).
4. *Cryopreservation of complex tissues and organs*: Extending cryopreservation techniques to complex tissues and organs remains challenging. Research is needed on improving methods to preserve vascularized tissues, whole organs, and organisms. Innovations in perfusion techniques and CPA delivery systems are key areas to explore. Protocols allowing effective banking of organs and vascularized composite tissues with full functional recovery post-cryopreservation will be highly beneficial to cryotherapeutics. This will enable a wide range of medical interventions to treat trauma and diseases in addition to enhancing transplant opportunities.
5. *Cryopreservation of stem cells and reproductive cells*: Cryopreservation of stem cells is critical for regenerative medicine. Methods to enhance the survival and functionality of stem cells post-thaw, including optimized CPA solutions and refined thawing protocols are relevant. More research will be appreciated in personalized cryopreservation or tailored protocols for individual patients to improve outcomes in cell-based therapies. Similarly, methods to improve the preservation, survival rates, and developmental potential of oocytes and sperm are critical.
6. *Optimal rewarming solutions for cryopreserved tissues and cellular repair post-cryopreservation*: The barriers towards the banking of organs and vascularized composite tissues can be solved by developing optimal rewarming solutions. Improving the cell repair processes post-thaw is a significant area of research. This includes developing better methods to mitigate ice damage, oxidative stress, and other injuries sustained during the cryopreservation process.
7. *Understanding cryobiology at the molecular level*: Advances in molecular biology and imaging technologies are helping scientists understand how freezing and thawing affect cells at a molecular level (Fahy and Wowk 2015; Baust *et al*. 2016; Taylor *et al*. 2019). This understanding can lead to better cryopreservation strategies and the development of new cryoprotectants.
8. *Long-term storage solutions*: For cryopreservation to be viable, solutions that can maintain cell integrity over extended periods need to be developed. This includes studying the effects of storage conditions and developing more stable cryopreservation protocols.
9. *Scalability and cost reduction*: Developing cost-effective methods will make cryopreservation more affordable and scalable, particularly for developing countries and small-scale applications.
10. *Integration with other technologies*: Exploring the integration of cryopreservation with emerging technologies such as artificial intelligence and genomic editing will enhance its applications and effectiveness.
11. *Ethical and regulatory considerations*: As cryopreservation technologies advance, addressing the ethical and regulatory considerations surrounding reproductive medicine and stem cell research is relevant. Engaging with the public and policymakers will help to develop supportive regulations and ethical guidelines that facilitate the responsible use and advancement of cryopreservation technologies. 

By focusing on these research directions, we can improve the efficacy and affordability of cryopreservation, unlocking new opportunities and driving progress across multiple sectors.

## Conclusion

Cryopreservation has evolved dramatically since its inception and has become an indispensable tool across a range of applications. It stands at the intersection of innovation and practical application, offering significant benefits across multiple sectors. Despite diverse applications in various realms, cryopreservation presents several challenges and opportunities for future advancements. Addressing these challenges requires ongoing research, innovation, and collaboration. Continued efforts in these areas will contribute to the advancement of cryopreservation technologies and their broader and responsible application in the future. Economically, this could make cryopreservation more accessible and cost-effective, potentially expanding its use and impact across various sectors. Exploring novel approaches such as nanotechnology and artificial intelligence in cryopreservation could further enhance its efficacy and applications. As we look to the future, the potential of cryopreservation is vast and promising. Embracing innovative solutions and fostering collaborative efforts will be crucial in realizing the full potential of this technology.
